# A wild-type *Botrytis* cinerea strain co-infected by double-stranded RNA mycoviruses presents hypovirulence-associated traits

**DOI:** 10.1186/1743-422X-10-220

**Published:** 2013-07-02

**Authors:** Christiaan A Potgieter, Antonio Castillo, Miguel Castro, Luis Cottet, Angélica Morales

**Affiliations:** 1 Deltamune (Pty.) Ltd., Lyttelton, Centurion, Lyttelton, South Africa; 2 Departamento de Biología, Laboratorio de Virología de Hongos, Facultad de Química y Biología, Universidad de Santiago de Chile, Casilla 40, Correo 33, Santiago, Chile; 3 Departamento de Ciencias Básicas, Facultad de Ciencias, Universidad Santo Tomás, Avenida Ejército Libertador 146, Santiago, Chile

**Keywords:** Mycovirus, Partitivirus, Double-stranded RNA, *Botrytis cinerea*, Transfection, Hypovirulence

## Abstract

**Background:**

*Botrytis cinerea* CCg378 is a wild-type strain infected with two types of double-stranded RNA (dsRNA) mycoviruses and which presents hypovirulence-associated traits. The objectives of the present study were to characterize the mycoviruses and investigate their relationship with the low virulence degree of the fungal host.

**Results:**

*B*. *cinerea* CCg378 contains five dsRNA molecules that are associated with two different types of isometric viral particles of 32 and 23 nm in diameter, formed by structural polypeptides of 70-kDa and 48-kDa, respectively.

The transfection of spheroplasts of a virus-free strain, *B*. *cinerea* CKg54, with viral particles purified from the CCg378 strain revealed that the 2.2-kbp dsRNAs have no dependency on the smaller molecules for its stable maintenance in the fungal cytoplasm, because a fungal clone that only contains the 2.2-kbp dsRNAs associated with the 32-nm particles was obtained, which we named *B*. *cinerea* CKg54vi378. One of the 2.2 kbpdsRNA segments (2219 bp) was sequenced and corresponds to the gene encoding the capsid protein of *B*. *cinerea* CCg378 virus 1 (Bc378V1), a putative new member of the *Partitiviridae* family.

Furthermore, physiological parameters related to the degree of virulence of the fungus, such as the sporulation rate and laccase activity, were lower in *B*. *cinerea* CCg378 and *B*. *cinerea* CKg54vi378 than in *B*. *cinerea* CKg54. Additionally, bioassays performed on grapevine leaves showed that the CCg378 and CKg54vi378 strains presented a lower degree of invasiveness on the plant tissue than the CKg54 strain.

**Conclusions:**

The results show that *B*. *cinerea* CCg378 is coinfected by two mycoviruses and that the 2.2-kbp dsRNAs correspond to the 32-nm mycovirus genome, which would be a new member of the *Partitiviridae* family as it has the typical pattern of partitiviruses. On the other hand, the results suggest that the hypovirulence of *B*. *cinerea* CCg378 could be conferred by both mycoviruses, since the fungal clone *B*. *cinerea* CKg54vi378 presents an intermediate virulence between the CKg54 and CCg378 strains. Therefore, the putative *partitivirus* would be partially contributing to the hypovirulence phenotype of the CCg378 strain.

## Introduction

Most of the currently characterized mycoviruses from yeasts and filamentous fungi are composed of one or more double-stranded RNA (dsRNA) molecules enclosed in an icosahedral or spherical isometric protein coat [1-3]. In filamentous fungi, mycoviruses with circular single-stranded DNA [4] and single-stranded RNA genomes [5-11], including unusual viruses without a true protein capsid, such as those of *Cryphonectria parasitica*, whose RNA genome is associated with lipid-rich pleomorphic membranous vesicles [12], are exceptions to this generalization. Nevertheless, several mycoviruses have been described that contain ssRNA, and it is very possible that their numbers will continue to increase [13]. Anyway, most of the described mycoviruses contain a genome that is predominantly composed of dsRNA, and they are classified into the *Totiviridae*, *Partitiviridae*, *Reoviridae* and *Chrysoviridae* families based on the number and size of their genomic segments [1,2].

The majority of the described virus-host systems correspond to single infections, i.e., only one mycovirus infects its host fungus [14]. However, mixed mycovirus infections in filamentous fungi by two or more unrelated viruses appears to be a relatively common phenomenon. Some more recent examples have been described in *Aspergillus fumigatus* isolates infected with more than one mycovirus, containing multiple dsRNA segments [15], co-infection of a hypovirulent isolate of the plant pathogenic fungus *Sclerotinia sclerotiorum* by two dsRNA mitoviruses [16], and mixed infection by dsRNA mycoviruses in black *Aspergillus* populations [17] and in *Fusarium graminearum *[18].

An important difference between mycoviruses and some traditional viruses of animals, plants and bacteria is that many mycoviruses are latent or cryptic, and they neither cause perceivable damage to their host fungus nor provide a particular phenotype by replication and/or expression of the viral genome [14]. However, there are some well-documented and extensively studied systems in which the presence of mycoviral dsRNAs conferred particular phenotypes to the host fungus. The killer systems of *Saccharomyces cerevisiae *[19] and *Ustilago maydis *[20] and the hypovirulence of *Cryphonectria parasitica *[21] are clear examples of this phenomenon.

*Botrytis cinerea* Pers. [teleomorph* Botryotinia fuckeliana* (de Bary) Whetzel] is a phytopathogenic fungus that infects a wide range of hosts, including ornamental plants, vegetables and fruits. This fungus causes the disease known as gray mold because the development of the fungal mycelium on the plant surface presents a velvety gray aspect. Some major fruits affected by this phytopathogenic fungus are strawberries, raspberries, pears, kiwis and grapes [22]. This fungus produces bunch rot in grapes, causing large pre- and postharvest losses. *B*. *cinerea* virology is a largely uninvestigated research field in which little information has been published [7,10,23-29]. Therefore, all new information related to *B*. *cinerea* mycoviruses can contribute to the understanding of virus-host interactions and to the design of strategies for the biological control of this phytopathogenic fungus.

This work describes a viral system in which a wild-type *B*. *cinerea* strain carries at least five dsRNAs that may represent dsRNA and/or ssRNA viruses.

## Materials and methods

### Fungal strains and culture conditions

Wild-type *B*. *cinerea* strains CCg378 and CKg54 were isolated in 1997 from rotten grapes from Rancagua, a city geographically located in the central zone of Chile. Both strains were grown at 20°C on 1.5% (w/v) malt extract, 0.7% (w/v) yeast extract (Merck Darmstadt, Germany).

### Double-stranded RNA purification

DsRNA was purified by cellulose CF11 chromatography, as described by Castillo et al., [30].

### Nucleic acid analysis, RNase A treatments, protein analysis, preparation and infection of spheroplasts of *B. cinerea* CKg54, sporulation rate determination, and virulence bioassays

All of these procedures were performed as described by Castro et al., [26].

### Isolation and purification of viral particles

Viral particles were isolated and purified as described by Castro et al., [25], except that in the last step, the particulate material was loaded into an equilibrium CsCl density gradient instead of a sucrose gradient and centrifuged for 38 hours at 30,000 rpm in a Beckman SW 55Ti swinging bucket rotor. The initial density was 1.34 g/mL. The gradient fractions were collected from bottom to top, i.e., the fraction with the greatest density corresponds to fraction 1.

### Electron microscopy

Negative staining of the viral particles and ultrathin mycelium sections were performed as described in Vilches and Castillo, [24]. Observations were performed on a Philips Tecnai 12 BioTWIN electron microscope at 80 kV.

### cDNA synthesis, cloning, sequencing and sequence analysis

The cDNA synthesis, cloning and sequencing was performed as described previously by Potgieter et al., [31]. The sequences of capsid protein of twenty-five partitiviruses, plus the sequence of the L-A totivirus [19] and the sequence of Bc378V1, were aligned using the software Clustal X [32]. To obtain phylogenetic relationships, alignments were analyzed by the neighbor joining method, using the MEGA 5.0 program [33]. The robustness of the branches generated by the analysis, were determined with 1000 bootstrap replicates.

### Laccase enzyme activity determination

The laccase activity was quantified spectrophotometrically using 2,6-dimethoxyphenol (DMOP) as substrate, as previously described [34].

## Results

### Characterization of dsRNAs in *B. cinerea* CCg378

**Figure 1:**
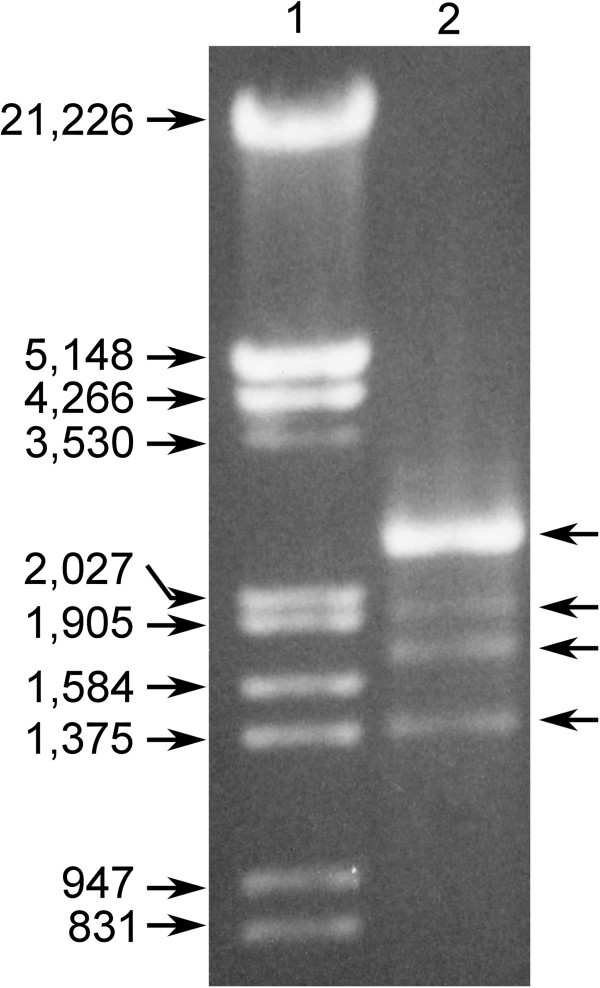
**Agarose gel electrophoresis of dsRNAs from *B*. *cinerea *CCg378.** Lane 1, lambda DNA/*Eco*RI + *Hin*dIII marker; lane 2, dsRNAs from *B*. *cinerea* CCg378 purified by CF11-cellulose chromatography. The numbers on the left indicate molecular sizes expressed in base pairs (bp). The arrows on the right indicate the position of dsRNAs.

The nucleic acid composition analysis of *B*. *cinerea* CCg378 revealed the presence of extrachromosomal genetic elements (EGEs) that had a faster electrophoretic mobility than that of genomic DNA. These molecules were sensitive to RNaseA treatment in a low ionic strength buffer, but they were resistant in a high salt concentration buffer (not shown). Furthermore, these genetic elements were retained in CF11 cellulose columns when an ethanol-containing solution was used as the elution buffer, but they were eluted with an ethanol-free buffer, which indicates that these molecules corresponded to dsRNAs. Agarose gel electrophoretic analysis of the CF11-cellulose-purified molecules showed four sharp bands of approximately 2.2, 1.95, 1.75 and 1.4 kilobase pairs (kbp) (Figure 1). The 2.2-kbp band had a considerably greater intensity than the smaller sized bands, and it contained two types of dsRNAs of almost identical molecular size that could be minimally separated by polyacrylamide gel electrophoresis (not shown).

### Purification and characterization of viral particles

The presence of viral particles associated with the dsRNAs was detected by preparing mycelium-free protein extracts, precipitating the particulate material and macromolecules with polyethylene glycol 8000, and purifying the mycoviruses in an equilibrium cesium chloride density gradient. Gradient fractions were dialyzed and analyzed using agarose gel electrophoresis to determine the nucleic acid content, imaged using electron microscopy after negative staining to visualize and characterize the viral particles, and evaluated using SDS-polyacrylamide gel electrophoresis to determine the structural polypeptide composition of the mycoviruses.

**Figure 2:**
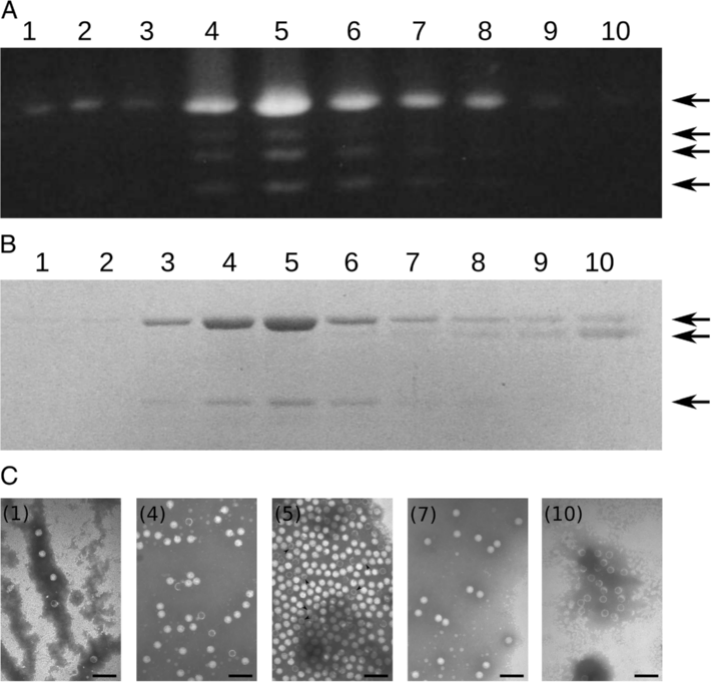
**Analysis of the gradient fractions from particulate material of *B*. *cinerea *CCg378, using 0.8% (w/v) agarose gel electrophoresis and 10% (w/v) SDS-PAGE. (A)** Nucleic acids present in gradient fractions 1–10. **(B)** Polypeptide profile of gradient fractions 1–10. The arrows on the right indicate the migration position of dsRNAs **(A)** and major polypeptides **(B)**. **(C)** Electron micrograph of mycoviruses from *B*. *cinerea* CCg378 present in representative gradient fractions. Viral particles of gradient fractions 1, 4, 5, 7 and 10 are shown. Arrows in **C** (5) indicate the position of some mycoviruses of 23 nm in diameter. Negative staining with 2% (w/v) potassium phosphotungstate, pH 7.0. The bar marker in 1, 4, 5, 7 and 10 represents 100 nm.

The nucleic acid electrophoretic profile detected in the gradient fractions is shown in Figure 2A. All gradient fractions presented the highest molecular weight dsRNA band, except fractions 9 and 10, where there was very little of this band and its presence was almost undetectable (Figure 2A). Four dsRNA bands were observed in fractions 4–8, and fraction 5 presented the highest intensity. Both the relative abundance and the types of viral particles correlated perfectly with the types of nucleic acids detected in the gradient fractions (see below).

**Figure 3:**
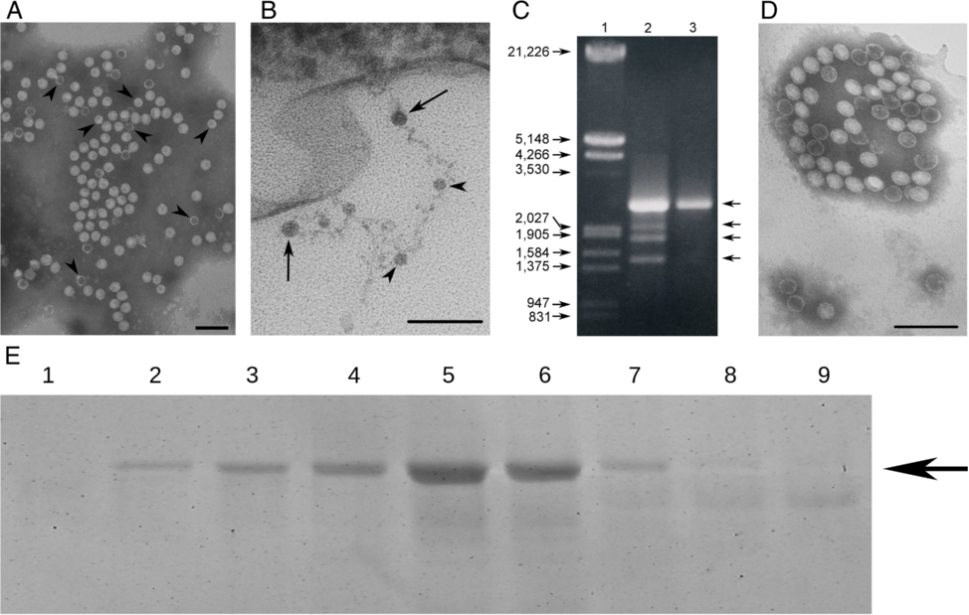
**Structural characterization of mycoviruses that infect to *B. cinerea *CCg378. (A)** Electron micrograph of mycoviruses from *B*. *cinerea* CCg378 present in gradient fraction 5. Arrowheads indicate the position of 23-nm viral particles. **(B)** Ultrathin section of *B*. *cinerea* CCg378 mycelium containing viral particles. Arrows and arrowheads indicate the position of some mycoviruses of 32 and 23 nm in diameter, respectively. **(C)** Agarose gel electrophoresis of dsRNAs from *B*. *cinerea* CKg54vi378. Lane 1, lambda DNA/*Eco*RI + *Hin*dIII marker; lanes 2 and 3, dsRNAs from *B*. *cinerea* CCg378 and *B*. *cinerea* CKg54vi378 purified by CF11-cellulose chromatography, respectively. The numbers on the left indicate molecular sizes expressed in base pairs (bp). The arrows on the right indicate the position of dsRNAs. **(D)** Electron micrograph of mycoviruses from *B*. *cinerea* CKg54vi378. **(A)** and **(D)** Negative staining with 2% (w/v) potassium phosphotungstate, pH 7.0. The bar marker in **(A)**, **(B)** and **(D)** represents 100 nm. **(E)** Polypeptide profile of gradient fractions 1–9 of particulate material obtained from *B*. *cinerea* CKg54vi378. The arrow on the right indicate the migration position of the major polypeptide.

Electron microscopy revealed that in the two first fractions a small amount of only one type of isometric viral particles were found, which were apparently icosahedral and 32 nm in diameter (Figure 2C, panel 1); however, in fraction 3, a very small amount of 23 nm particles was detected in addition to the 32-nm particles. In fractions 4–8, proportionally larger amounts of both types of isometric viral particles, i.e., 32 nm viral particles identical to those found in fractions 1–3 and 23 nm particles identical to those found in fraction 3, were detected (Figure 2C, panels 4, 5 and 7). In fractions 9 and 10, although not very abundant, all of the observed viral particles were empty capsids of 32 nm in diameter (Figure 2C, panel 10). As shown in Figure 3A, it is possible to distinguish both types of viral particles and estimate the approximate proportions of each type in the samples.

The SDS-polyacrylamide gel electrophoresis analysis revealed that there was only a very low concentration of one major polypeptide that was approximately 70 kDa in fractions 1 and 2 (Figure 2B, lanes 1 and 2); in fractions 3–8, two major polypeptides of 70 and 48 kDa were present (Figure 2B, lanes 3–8); and in fractions 9 and 10, two polypeptides of 70 and 65 kDa were detected (Figure 2B, lanes 9 and 10). These results suggest that the 70 kDa band corresponds to the main structural polypeptide of the 32 nm viral particles and that the 48 kDa band corresponds to the major capsid polypeptide of the 23 nm viral particles.

### Subcellular location of viral particles

Electron microscopy of the ultrathin mycelium sections of *B*. *cinerea* CCg378 revealed the presence of two types of isometric virus-like particles that were approximately 32 and 23 nm in diameter and that had similar morphologies as those purified by CsCl density gradient centrifugation (Figure 3B). The mycoviruses were generally found to be associated with membranous cytoplasmic structures, and it was possible to observe long filamentous structures, likely the viral nucleic acids, associated with the viral capsids (Figure 3B). These results indicate that *B*. *cinerea* CCg378 is a pure fungal strain co-infected by two different mycoviruses rather than two different fungal strains that are each infected by different viruses.

### Transfection of spheroplasts of *B. cinerea* CKg54 with viral particles purified from *B. cinerea* CCg378

In an attempt to separate both types of viral particles and to determine if the low virulence of the CCg378 strain is conferred by the mycoviruses that it hosts, spheroplasts of a virus-free strain, *B*. *cinerea* CKg54, were transfected with purified viral particles (fraction 5 of the gradient, Figure 2C, panel 5) and the dsRNA contained in the monosporic clone obtained was analyzed. Several isogenic fungal clones infected only with the 32 nm mycovirus and containing only the dsRNAs of approximately 2.2 kbp and the 70 kDa capsid polypeptide were obtained (Figure 3C, D and E). Clones containing the 23 nm virus or the dsRNAs of lower molecular size were not found. For later analyses, a monosporic clone infected with the 32 nm virus named *B*. *cinerea* CKg54vi378 was selected (Figure 3C, D and E).

### Sequence analysis

The dsRNA band of Bc378V1 of about 2.2 kbp can be resolved by polyacrylamide gel electrophoresis and was composed of two separate genomic segments of almost identical molecular size. The complete nucleotide sequence of the cDNA of one of them (dsRNA of 2219 bp; GenBank accession number KF201714) exhibits similarity with the capsid proteins of fungal partitiviruses. The putative coat protein segment consisted of an ORF of 1902 nucleotides, coding for a polypeptide of 634 aa (Mr 70379.59 Da), flanked by a 5′ UTR of 117 bp and 3′ UTR of 113 bp, including 57 adenines with a great concentration of them to the sequence end.

**Figure 4:**
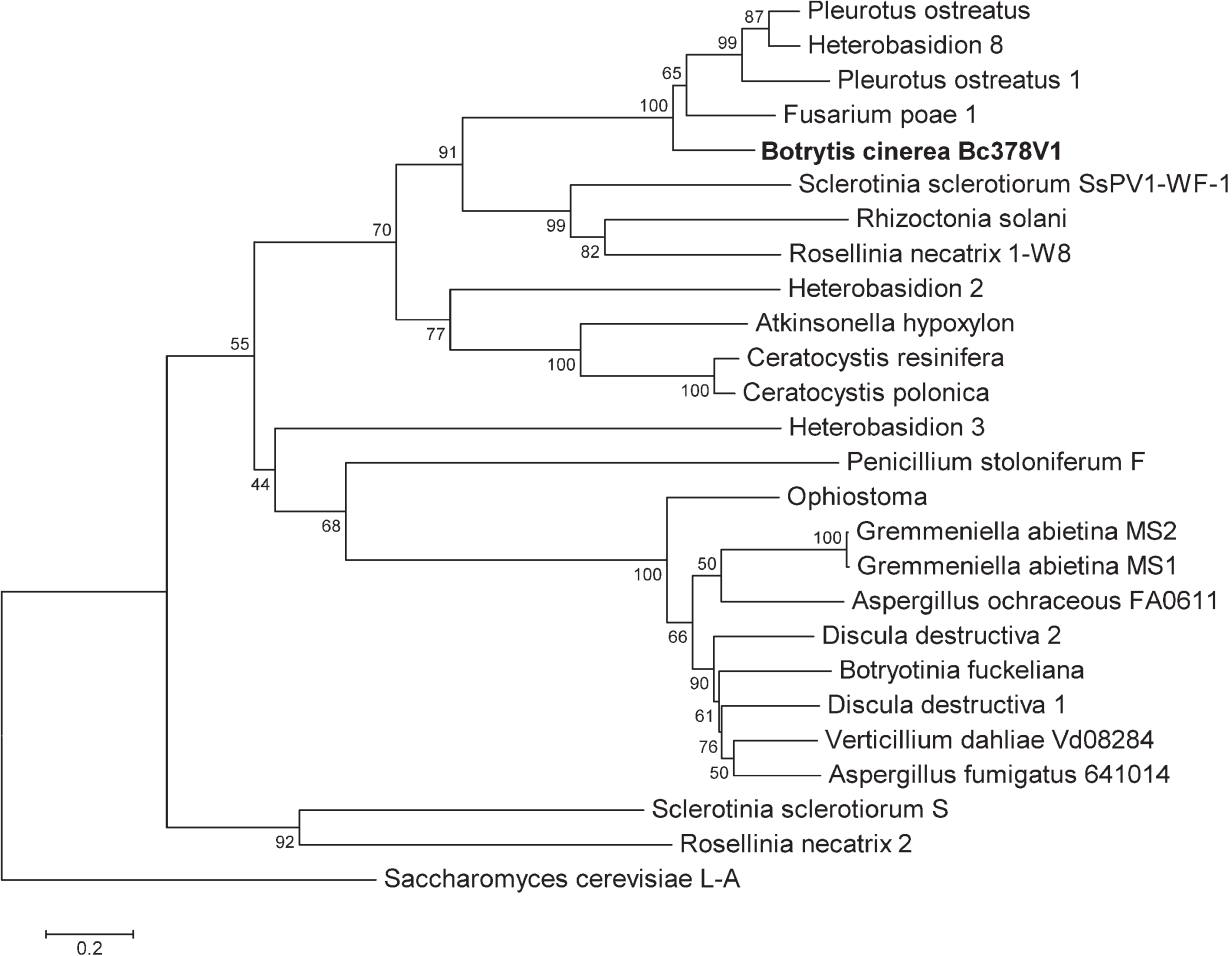
**Phylogenetic tree generated from viral coat protein alignments of multiple members of the family *Partitiviridae *and Bc378V1.***Saccharomyces cerevisiae* L-A totivirus [19] was added as an outgroup. Bc378V1 is indicated in bold.

The deduced amino acid sequence of the capsid protein segment was used for phylogenetic analyses based on alignments of the amino acid sequence of the coat protein of members of the *Partitiviridae* family and of Bc378V1 capsid protein (Figure 4). MEGA 5 software [33] was used to estimate genetic distances and to make a neighbour-joining tree. As expected, Bc378V1 was included in a clade within the partitiviruses, specifically with the *Fusarium poae* dsRNA mycovirus 1 [35].

### Virulence parameter analysis

**Figure 5:**
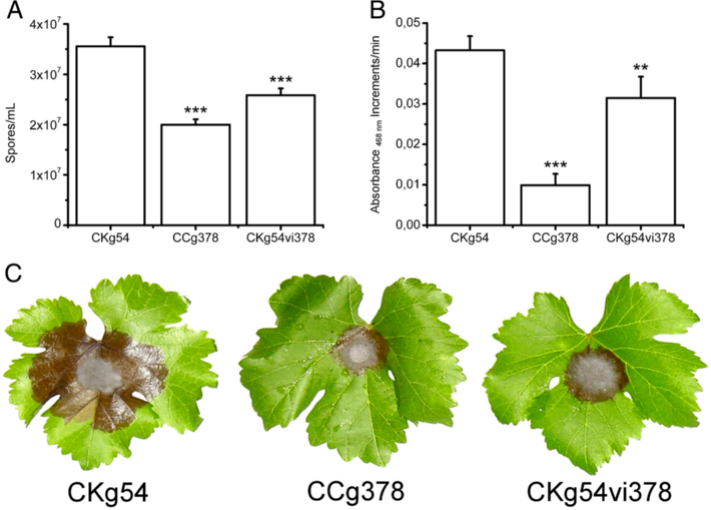
**Comparison of physiological parameters associated with the virulence degree in *B*. *cinerea *CCg378 (co-infected strain), *B*. *cinerea *CKg54 (virus-free strain) and *B*. *cinerea *CKg54vi378 (clone infected with the 32-nm virus only). (A)** Spores production, average of five experiments. **(B)** Laccase activity quantitation, average of five experiments. **(C)** Comparison of the virulence degree of *B*. *cinerea* CKg54, *B*. *cinerea* CCg378 and *B. cinerea* CKg54vi378. The fungal strains were inoculated in the center of grapevine leaves. The brown zone on the leaf corresponds to the lesion caused by the fungus. Data were analyzed by one-way ANOVA with Bonferroni post-test, ** represents significant differences at p <0.01, and *** represents significant differences at p <0.001.

The sporulation rate and laccase enzyme activity were assessed as the *B*. *cinerea* virulence parameters. The *B*. *cinerea* CCg378 virulence parameters were compared with those of *B*. *cinerea* CKg54, a wild-type strain that lacks extrachromosomal genetic elements, and with the infected monosporic clone, *B*. *cinerea* CKg54vi378. Figure 5A shows the comparison of the conidiation rate. *B*. *cinerea* CKg54 had a conidiation rate that was approximately 1.75-times greater than that of *B*. *cinerea* CCg378 and 1.40-times greater than that of *B*. *cinerea* CKg54vi378. Quantitative determination of the laccase activity was performed spectrophotometrically using 2,6-dimethoxyphenol as a substrate. The laccase activity was lower in the CCg378 strain and intermediate in the CKg54vi378 clone compared with the virus-free CKg54 strain (Figure 5B).

### Virulence bioassays

The above results correlated with the virulence bioassays performed on grapevine plant leaves, in which the CKg54 strain was highly aggressive against the vegetable, while the CCg378 and CKg54vi378 strains presented low and intermediate invasiveness profiles, respectively (Figure 5C). Therefore, the virulence parameters (sporulation rate and laccase activity) and bioassays indicate that the CCg378 strain is a hypovirulent strain.

## Discussion

This is the first report that describes the existence of a wild-type *B*. *cinerea* strain simultaneously infected with two different viral particles types that are possibly related to the hypovirulence of the host fungus. One of the mycoviruses would be a putative partitivirus because of its morphology and particle size, the molecular mass of its capsid protein, its possession of a dsRNA bipartite genome in the molecular size range of members of the *Partitiviridae* family and its high capsid protein sequence similarity with *Fusarium poae* dsRNA mycovirus 1. In this context, the initial observations were supported by the fact that the CsCl gradient fractions 1 and 2 contained only particles of 32 nm in diameter associated with dsRNAs of 2.2 kbp. These results confirm that these viral particles contain two dsRNA molecules of approximately 2.2 kbp with identical electrophoretic migration in agarose gel, minimal separation in polyacrylamide gel, and most likely different nucleotide sequences, with one of them encoding for the 70 kDa polypeptide (capsid protein) and the another one for the viral RNA-dependent RNA polymerase (RdRp) [1].

In fractions 3–8, two types of mycoviruses (32 and 23 nm in diameter) were observed to be associated with five dsRNA molecules. The larger particles (32 nm) were considerably more abundant than the smaller particles (Figure 2C, panel 5 and Figure 3A), which correlated perfectly with the amount of dsRNAs detected in these fractions. In fact, the densitometric analysis of the nucleic acid bands in fractions 4–6 showed that the larger dsRNA band was present in an amount at least five-times greater than that of the sum of all the smaller dsRNAs. At the same time, the 70 kDa polypeptide band was also found in a quantity at least five-times greater than that of the 48 kDa polypeptide. How the virus-host interaction regulates the stoichiometry of both viral particle types is unknown. We do not have a clear explanation for the appearance of the polypeptide band of approximately 65 kDa in fractions 7–10. This band may be a proteolytic fragment originating from the degradation of the 70 kDa polypeptide.

By directly visualizing ultrathin mycelium sections of the fungus with electron microscopy to determine the subcellular location of mycoviruses, we were able to detect both types of viral particles in the same fungal cell (Figure 3B), which is very solid evidence for the co-infection of *B*. *cinerea* CCg378 by two different mycoviruses.

Because the intracellular quantity of 32 nm viral particles was considerably greater than the quantity of 23 nm particles in the CCg378 strain, we expected to obtain monosporic clones infected only with the 32 nm virus and containing only the 2.2 kbp dsRNAs when a virus-free fungal strain was transfected with purified viral particles obtained from fraction 5 of the CsCl gradient. Our results with the above mentioned fungal clones demonstrate that it is possible to separate the 32-nm mycovirus and that its stable intracellular maintenance does not depend on the presence of the 23 nm particles or the smaller dsRNA molecules (Figure 3C and D).

The physiological parameters associated with the virulence, laccase activity and sporulation rate of the CCg378 strain were the lowest compared to those of *B*. *cinerea* CKg54, a virus-free strain, and *B*. *cinerea* CKg54vi378 (a clone infected only with the 32-nm mycovirus). Suppressed laccase activity is a common phenotypical change associated with hypovirus infection in *C*. *parasitica *[36,37] and *Diaphorte ambigua *[34]. It has been suggested that laccase secreted by *B*. *cinerea* may act as a detoxifying enzyme to protect the fungus from the toxic metabolites formed during pathogenesis and to reduce the host’s lignification activity [38]. In fact, *B*. *cinerea *laccase is able to degrade pterostilbene and resveratrol, two phytoalexins from grapes [39]. In addition, it has been shown that the repression of laccase production transforms *B*. *cinerea* into a weak pathogen [38], confirming the importance of this enzyme in the virulence of this fungus [40]. Both the laccase activity and sporulation rate are physiological parameters that have been used as virulence parameters in other dsRNA-containing phytopathogenic fungi [34,41,42].

Our results show that *B*. *cinerea* CCg378 is infected by two different dsRNA mycoviruses. Experimental evidence suggests that the genome of the 32-nm mycovirus consist of the two dsRNAs of about 2.2 kbp. Furthermore, it is known that the monocistronic dsRNAs of the partitiviruses are encapsidated separately in identical capsids, one of them containing the dsRNA encoding the capsid protein and the other the dsRNA encoding the RdRp [43]. We presented confirmatory evidence that the 32 nm mycovirus is a new member of the partitiviruses, since the sequence analysis of the cDNA corresponding to the gene of the capsid protein showed high similarity with members of the *Partitiviridae* family. On the other hand, the genome of the 23-nm mycovirus would correspond to one, two or the three smaller dsRNA molecules; however, due to the small size of the virion, they should be separately encapsidated. Another possibility is that some of the smaller dsRNAs correspond to a satellite dsRNA or to defective-interfering dsRNA (DI-dsRNAs), a common feature of mycovirus infections [1]. *Atkinsonella hypoxylon*, *Discula destructiva*, *Gremmeniella abietina* and *Rosellinia necatrix *[43,44] are some fungal species infected with partitiviruses in which satellite dsRNAs or DI-dsRNAs have been detected. Therefore, it is clear that the genome composition of the 23-nm mycovirus remains to be elucidated.

Finally, our results suggest that the low virulence of the CCg378 strain may be partially caused by the presence and/or gene expression of the putative 32-nm partitivirus because the infected clone CKg54vi378 presents an intermediate virulence between that of the CKg54 and CCg378 strains. Therefore, the complete hypovirulence phenotype would be conferred by the presence of both mycoviruses and/or by the expression of both mycoviral genomes with the consistent generation of gene products that regulate the attenuation of host fungus virulence at the molecular level.

## Conclusions

*B*. *cinerea* CCg378 is a wild-type strain co-infected by two dsRNA mycoviruses. The genome of the 32-nm mycovirus consists of two dsRNA molecules of about 2.2 kbp each, and its capsid is formed by a 70-kDa structural polypeptide. The molecular composition of the 23-nm mycovirus remains to be elucidated. The phenotype of hypovirulence that *B*. *cinerea* CCg378 presents would be conferred by both mycoviruses, because the fungal clone *B*. *cinerea* CKg54vi378, infected only with the 32 nm mycovirus, presents an intermediate virulence between a virus-free fungal strain, *B*. *cinerea* CKg54 and *B*. *cinerea* CCg378.

## Competing interests

The authors declare that they have no competing interests.

## Authors’ contributions

CAP performed the cDNA synthesis, cloning and sequencing experiments; AC designed the study, performed the electron microscopy experiments and wrote the manuscript; MC participated in the design of experiments and carried out the molecular characterization of viral particles; LC participated in the design of experiments and performed the phylogenetic analysis and determinations of virulence parameters of fungal strains; AM participated in the design of experiments and carried out the transfection experiments and virulence bioassays. All authors read and approved the final manuscript.
